# Re-Investigation on Periodic Assembly in Crystallized Poly(ethylene adipate) by Dissecting into Internal Architecture

**DOI:** 10.3390/polym18131666

**Published:** 2026-07-06

**Authors:** Chi-Hsuan Su, Selveraj Nagarajan, Chean-Cheng Su, Eamor M. Woo

**Affiliations:** 1 Department of Chemical Engineering, National Cheng Kung University, Tainan 701-01, Taiwan; washownswii@hotmail.com (C.-H.S.); nagarajan.tech@gmail.com (S.N.); 2 Department of Chemical and Materials Engineering, National University of Kaohsiung, Kaohsiung 811, Taiwan; ccsu@nuk.edu.tw

**Keywords:** poly(ethylene adipate), periodic band, crystal assembly, surface-relief pattern, discontinuity

## Abstract

Through microscopy analyses onto 3D-dissected interiors of crystallized poly(ethylene adipate) (PEA) at isothermal 28 °C temperatures that are known to pack with double ring-banded spherulites, complete surface-relief patterns correlating with interior periodic assembly profiles on mechanisms are obtained. Top-surface-relief ridge bands exhibit different width caused by slant angles between the interior radially orientated lamellae with respect to the top surface. The detailed interior structures gained from analyses on the dissected PEA spherulites yield critical correlations between the interior assembly and topology banding patterns, leading to a clue that analyses cannot be restricted to simply just on the top-surface-relief patterns. With advanced dissection techniques, 3D views on bulk interiors have offered dramatic breakthrough views and led to unique clarity in assembly mechanisms of periodic crystal aggregation.

## 1. Introduction

Similar to the self-organized crystal growth observed in confined colloidal systems [1,2], crystallization of materials takes place in gradually higher hierarchical orders from lattice cell/units (nanometers) to intermediate lamellae and finally into micrometer-size aggregated spherulites. Varieties of geometric assembly of lamellae have been known to lead to final spherulites showing either ring bands such as poly(ethylene adipate) (PEA) or non-ring-band poly(butylene terephthalate) (PBT) [3]. Other optical-birefringent patterns, such as leaf-like [4], sheaf-like [5], and dendritic [6], have also been common. Crystal assembly usually takes place in fractal patterns that self-repeat or are like those seen in snowflakes in dimensions from micrometer to millimeter. Ring-banded morphologies can be classified as (a) zero-birefringence (concentric ring-bands) [7,8], (b) single-interference color-bands with extinction, and (c) double-interference bands of positive- and negative-type birefringence colors in spherulites alternately coexisting, such as banded spherulites in an arylate polyester of poly(nonamethylene terephthalate) (PNT), or quadrant-specific spherulites with alternating banded and non-banded sectors [9]. Variation in types of ring-banded morphologies might be caused by different mechanisms of crystal growth and assembly formation and should be analyzed from different perspectives. Various models for the ring-banded morphology have been continuously probed for better proofs for almost half a century, with the existing ones periodically re-visited for refinement.

Double-helical DNA molecules, firmly held in stable conformation via millions of pairs of intramolecular H-bonding, were first proven by Nobel-prize laureates Watson and Crick in 1953 [10]. Since then, many scientists working on polymer crystals, such as Keller [11,12,13,14], Keith and Padden [15,16,17,18,19], Bassett and Hodge [20,21,22,23], had extensively investigated olefin polymers, such as polyethylene (PE), proposing tentative interpretations by mimicry of the tips of proven helical-twist conformation in DNA molecules. It then became a popular concept that the ring-banded morphology of periodic optic bands might be also expounded by similar models of continuous lamellae that helical twist and extend outward from a common center. They attempted to depict each of the helicoid lamellae by assuming a straight helix geometry without any branches. However, the molecular structures of deoxyribonucleic acid (DNA) with a double helix that is known to be stabilized and held by many intra-molecular H-bonding sites [10] are absent in the chemical chains of most polymers that display periodic bands in spherulites. Since the 1950s, the early pioneering investigators and many recent ones have proposed for the past several decades that lamellae continuously twisting was responsible for the formation of optical periodic banding in ring-banded spherulites of many different polymers [24,25,26,27,28]. By re-visiting the classical models, Lotz and Cheng [24] in 2005 had proposed that the repeated chain folding in the lamellae of long polymer chains is the main cause of inducing the surface stresses that are needed for maintaining continuous “lamellae helical-twist” in banded polymer spherulites. These classical propositions had been frequently re-visited and comprehensively investigated, yet it remained highly debated and questioned as there had been no direct and solid evidence for viewing the interior nano- or micro-architectures of the periodic assembly in polymer spherulites.

In the fields of polymer crystals, the lamellae are thought to have a chain-folding nature in assembly into crystal lamellae, thus, inducing surface stresses. Outside the polymer arena, by contrast, the classical literature shows ample examples that spherulites of small-molecule organic compounds (such as phthalic acid, PA) or inorganic mineral compounds (such as K_2_Cr_2_O_7_) form similar ring bands. Unlike the long-chain polymers, these small-molecule crystalline compounds are known to lack any chain folding in the assembly of crystals [29,30,31,32,33,34]. Among the evidence presented in the literature from time to time in the past half a century [35,36,37,38], there is discrepancy between the dimensions for lamellae helical-twist pitches (observed in SEM data) versus the optical inter-band spacing (observed in optical polarized-light microscopy) in polymer-banded spherulites. Thus, scientifically, the issues of classical models of continuous helical-twist lamellae remain highly debated and cannot be satisfactorily settled.

In contrast to most classical investigations analyzing mainly on the topology of thin films, our more recent work pioneered into newer frontiers by digging deeper into the inner bulk assembly of banded polymer spherulites (rather than being confined in thin films) [39]. This work was further extended by using PEA as a model, and dissected the 3D interiors with wider scopes of analyses by correlating the top-surface optical patterns and surface banding morphology with the interior crystal assembly mechanisms. Furthermore, this work re-investigated the model system to shed newer light on interpreting how and why the proposed assembly mechanisms could be justified with solid evidence to be discussed. New frontiers are urgently necessary in constructing more workable mechanisms of lamellae assembly in complex spherulites displaying periodic patterns; thus, the objective of this study was to use the widely studied PEA spherulites with orderly dual-birefringence periodic patterns as an ideal model for understanding the details of PEA-banded spherulite interior structure in 3D point of view, and correlation of the top-surface and fractured interiors in 3D perspectives, which were correlated to the top-surface morphology patterns.

## 2. Experiment

### 2.1. Materials and Preparation

PEA was obtained from Aldrich Chemicals, with T_g_ = −52 °C, T_m_ = 47 °C, and M_w_ = 10,000 g mol^−1^. Thin-film samples were cast by THF solution on glass micro-slides at 45 °C, while thicker ones were drip-cast with polymer solution several times onto micro-slides. Table 1 lists the sample preparation methods to achieve various film thicknesses used in this study. The film thickness was controlled by varying the polymer concentration and casting volume. The thickness was measured using a Sylvac μS229 digital thickness gauge (SYLVAC SA, Yverdon-les-Bains, Switzerland), with measurements obtained at multiple locations on each film. The reported values represent the thickness range obtained from independently prepared samples, demonstrating good reproducibility. Crystallization of samples was performed at T_c_ = 28 °C (on a temperature-controlled microscopic hot stage). This isothermal temperature T_c_ = 28 °C was selected because it led to the best regularity of bands.

### 2.2. Procedure and Apparatus

Polarized-light optical microscopy (POM, Nikon Optiphot-2, Tokyo, Japan) was used for birefringent patterns. A Nikon Digital Sight (DS)-U1 camera control system was used for the imaging process. In situ temperature control of specimens was performed using a microscopic hot stage (Linkam THMS-600 with T95 temperature programmer, Surrey, UK) designed for microscopy.

Scanning electron microscopy (SEM, Hitachi-SU8010, HR-FESEM, Tokyo, Japan) was used for microscopy characterization. Samples were vacuum-sputter coated prior to SEM characterization. Interior bulk morphology was probed using a delicately manipulated technique of fracturing the specimens to expose the inner architectures that were hidden beneath the top surface.

## 3. Results and Discussion

### 3.1. Topology and Birefringence Characteristics of Banded Architectures

**Figure 1:**
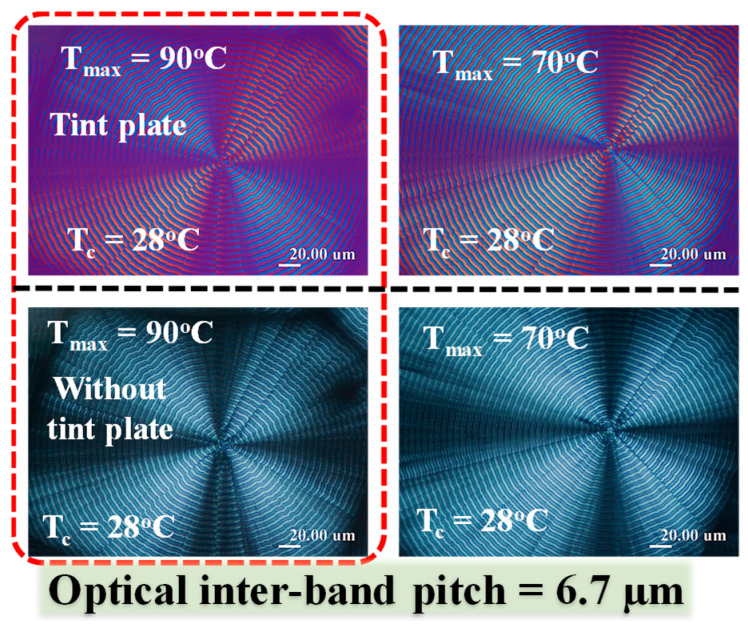
POM images for PEA at T_c_ = 28 °C, by quenching from two different levels: T_max_ = 70 °C and 90 °C (T_max_–90 °C), with or without tint plate (top vs. bottom), The images corresponding to T_max_ = 90 °C are highlighted by the red dashed outline.

Interior morphology of brightly birefringent dual-banded PEA spherulites was first examined at low-magnification for viewing the entire profiles, where sample films would be fractured across the films secured on glass substrates. Figure 1 shows four POM micrographs to reveal the birefringence patterns of PEA at T_c_ = 28 °C, by quenching from four different levels of T_max_ (90 and 70 °C). For comparison, both birefringence patterns with or without a tint plate are placed on the top and bottom, respectively. The tint plate induced coloration (blue vs. orange) for ease of inspecting the alternate rings with opposed crystal orientations.

**Figure 2:**
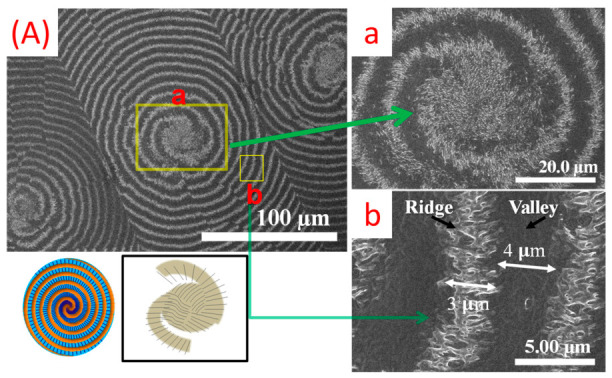
Top-surfaces bands in PEA: (**A**) SEM images of top surface bands in PEA spherulites showing variation of width of ridge and valley regions, (**a**) zoom-in to core, (**b**) zoom in to periodic bands, The two schematic illustrations in the lower-left corner depict the spiral ring-band morphology and the corresponding lamellar assembly model.

Samples of PEA crystallized at T_c_ = 28 °C with different film thicknesses were analyzed for evaluating the effect of geometry confinement. Results are shown in Figure 2. The nucleus center is dominated with spiral spins, which evolve into the periodically banded PEA spherulites. The inter-band spacing remains ca. ~7 µm, but the relative width of the respective ridge and valley regions could decrease and increase, respectively. Apparently, without morphology analysis by dissecting into the interiors, it would be difficult to understand the assembly details of the lamellae packed into an aggregated spherulite featured with periodically alternate bands.

From the above SEM graphs, the nucleus core initiates rings that go into spirals or Archimedean double spirals. Periodic rings can be classified into three kinds (circular rings; single spirals of either clock-wise spin or counter-clockwise spin, and double spirals). In general, the actual patterns on top views may involve three possibilities: (c-1) concentric circular rings, (c-2) single Archimedean spirals from a single end of nuclei, and (c-3) dual spirals originating from both ends of the nuclei. Apparently, what one views on the crystals from the top surface may differ from the interior view on crystals that actually are stacked directly underneath the top-surface lamellae assembled as Archimedean spiral bands. The experimental evidence of variety patterns of spiral spins, and not necessarily concentric circular rings, suggests that the periodic morphology in periodically-banded polymeric PEA spherulites may not be constructed by continuous helicoid-shape lamellar plates simultaneously radiating out from a common nucleus center. Instead, the rings can be composed of lamellae that are initiated from two ends of nucleus sheaves. This is a view from top-surface-relief patterns; more critically, 3D interior dissections will give more clues, as discussed in the next sections.

### 3.2. Variation of Inter-Band Spacing and Periodic Discontinuity Along the Radial Direction

**Figure 3:**
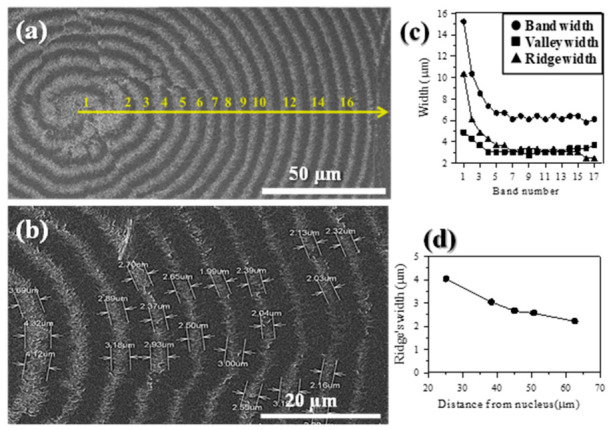
(**a**,**b**) SEM graphs for banding pattern of PEA spherulites, In (**a**), the yellow arrow indicates the radial growth direction, and the numbered labels denote successive ring-band cycles counted outward from the nucleus, and (**c**,**d**) ridge width decreasing with respect to distance from the nuclei core, with the valley width staying roughly constant.

The inter-band spacing is not uniform and may vary steadily with the distance from its nucleus core due to variation of kinetics-driven growth. In general, near the nucleus core, the molten species are relatively more abundant, and thus, the inter-band spacing tends to be larger. Oppositely, farther away from the nucleus core, the molten species are more drained out; thus, the inter-band spacing becomes stable and then smaller and less regular near the exterior rim. The variation in width of the ridge vs. valley regions was quantitatively estimated as a function of distance from the central nuclei core. This periodic feature is similar to the classical Liesegang rings that are known to be driven by reaction kinetics modulated by gels. For growth of banded spherulites, it is driven by crystallization kinetics, similarly modulated by super-cooled molten species surrounding the growing crystals. Figure 3 shows (a,b) two SEM micrographs for top-surface-relief patterns of banding in PEA spherulites, and (c) plots of width of ridge, valley, and entire band regions, as functions of the numbered bands, where the bands are numbered from #1 to #16 from nucleus core to periphery (spherulitic radius being ~120 µm). The inter-band width is wider with some irregularity near the nucleus center of PEA-banded aggregates, which gradually reaches a constant plateau value of inter-band spacing d = 6–7 µm. Note that near the core, the inter-band distance can be nearly twice as large (~10–15 µm) and rings near the core appear to take a shape of Archimedean spirals (Left center of Figure 3a). By comparison, Figure 3b shows that at farther distances from the nucleus core, the interior spheroid shells (underneath the top-surface bands) intersect with the top surface at a decreasing angle, which accounts for larger ridge width near the core but narrower and finer ridge width on the top-surface-relief pattern. The width of the entire band (ridge + valley), the ridge, and valley zones are estimated, as shown in Figure 3c. Their widths generally decrease from the nucleus core to a plateau value. For greater magnification of ridge width decreases upon near the periphery of the spherulite, Figure 3d shows the ridge width corresponding to distance of the bands (between 25–65 µm) from the core. By comparison, within this range of distance from the core (25–65 µm), the width of the valley region, owing to being the submerged lamellae, stays pretty much the same at 5 µm, regardless of distance from the core. By comparison, the width of the ridge (protruded portion of lamellae) is less at ca. ~2 µm. The varying pitches (inter-band spacings from nucleus to periphery) would also make it hard to justify that the lamellae continuously helical twist from a common center, as the double-helix DNA molecules are held by millions of pairs of base units with hydrogen bonding, and thus display a fixed pitch of helices. Furthermore, it would be impossible to justify that the periodic bands were caused by conventional thoughts of chain-folding-induced “surface stresses”, when the experimental fact is that the inter-band spacing is not a constant but varies dramatically. The inter-band spacing would have maintained a statistically reasonable constant if chain-folding-induced surface stresses were the responsible origins.

Obviously, the inter-band spacing does not maintain a constant as the lamellae of spherulite grow from the nucleus center into periodic bands, but rather it drops dramatically from the nucleus center from 15 µm to stabilize at ca. 6.5 µm as the ring number reaches 5 or beyond. The neighboring spherulites at full crystallinity start to impinge upon each other as the ring number matures at 16 or higher. The nucleation sheaf-crystals usually occupy a certain wider space; only away from the nucleus region do the periodic bands start to pack. Film thickness of specimens also influences the ridge/valley width, as the angles of intersecting between the interior spheroidal shell layers with the top surface may vary with film thickness. In the thinner films (3–10 µm), most of the interior lamellae are confined to a more limited range of inclination of angle with respect to the top/substrate surface; thus, the ridge/valley width on surface-relief banding patterns varies relatively less. By contrast, the lamellae in thicker PEA samples (>30 µm) have a wider range of angle of lamellae inclination compared to the thinner samples, allowing analyses on correlation between the distance from nuclei center and the ridge width. The area of the core region of the banded PEA spherulite also increases with film thickness, which all suggest that the diameters of the interior shell-layered spheroids evolving from interior sheaf-like nuclei sensitively influence the surface-relief patterns (core size, width of ridge vs. valley, etc.).

**Figure 4:**
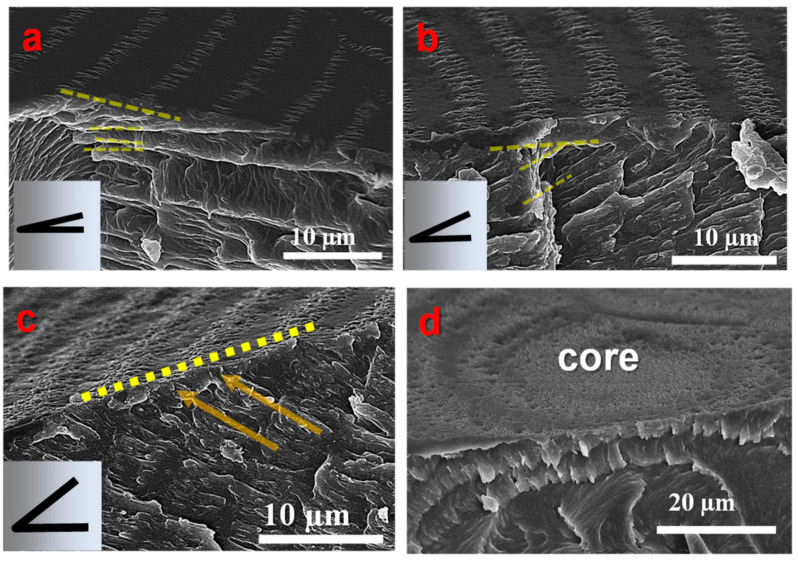
SEM micrographs comparing the fractured interiors with the corresponding top surfaces of PEA spherulites. (**a**–**c**) The images reveal that the interior lamellae intersect the top planar surface at oblique angles rather than normal to the surface. Yellow dashed lines delineate the lamellar orientation observation on the top surface, whereas yellow solid arrows highlight the inclination and growth direction of the fractured interior lamellar stacks. (**d**) Fracture through the spherulite core, illustrating the nucleation center and the emergence of the alternating banded lamellar architecture. Insets denote the fracture direction.

The interior lamellar assembly beneath the top-surface relief periodic patterns is key for understanding the mechanism. As the top-surface banding patterns may be composed of lamellae coming from interiors, the lamellar assembly underneath the surface bands needs to be analyzed. Figure 4 shows SEM micrographs of the zoomed-in fracture surface vs. top-surfaces of PEA spherulites generated from bulk samples with different angles between protruded crystal lamellae and the top planar surface. The radial lamellae, intersecting with the tangential lamellae at a nearly 90° angle, comprise the valley region. Figure 4a,c show interior lamellae whereby the greater the slant angle of the radial lamellae, the narrower the valley region on the top surface; and vice versa, the smaller the slant angle, the wider the valley region. By similar mechanisms, the greater the slant angle is between the radial lamellae with the top surface, the rougher and wider the ridge region is (at the sacrifice of the narrower valley regions). Nevertheless, the total shell thickness of one layer is the sum of the ridge plus valley rings, remaining almost constant at ~7.0 µm. Figure 4d shows that at the center of the central core where no bands are visible (spiral-spin bands start to assemble at the outer rim of the nucleus core), the lamellae grow almost upright; thus, most of the interior lamellae are vertically outward in this nucleus core region.

**Figure 5:**
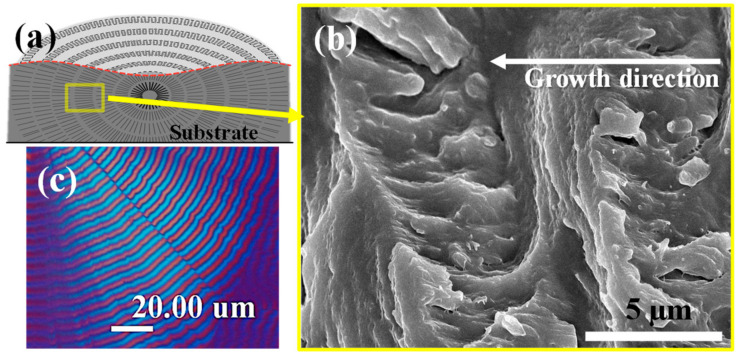
Cyclic repetition of growth with hierarchical assembly in discontinuous layers. (**a**) Scheme for top vs. interior of a banded PEA, red dash line separate top and interior, and (**b**) SEM zoom-in to blocked zone for grating-assembled interior, (**c**) POM micrograph of PEA.

By tracing and focusing on a single fully-grown PEA spherulite (at T_c_ = 28 °C), the interior PEA lamellae/crystals were dissected to reveal mechanisms in how the spherulite was packed into double ring-banded optical birefringence patterns. Figure 5a is a scheme depicting the SEM results for a whole banded PEA spherulite, where the morphology of fractured interiors is displayed to correlate with that of the top surface. As shown, PEA exhibits a multi-layered hierarchical structure indicating discontinuity between subsequent bands in the spherulite growth development. Figure 5b is a zoom-in high-magnification SEM graph, where the clean cleavage between the successive interior shelled layers reveals inter-band interfaces upon exposure of the lamellae packed in the interiors. Note that the crossbar pitch (i.e., inter-layer thickness) is ca. 6.7 µm, which perfectly matches with the optical inter-band spacing. The top-surface may appear to be superficially continuous and connected; yet, discontinuity between the successive bands is evident upon exposure by dissection into the bulk. The crossbar pitch, as revealed by SEM result, for the interior grating of ca. 6.7 µm, is exactly equal to that seen in optical inter-band spacing (Figure 5c—digitally enlarged POM to reveal the inter-band spacing). Discontinuity of the lamellae in neighboring bands is obvious and they are periodically assembled as a cross-hatch grating architecture in the interior bulks of the PEA-banded spherulites.

**Figure 6:**
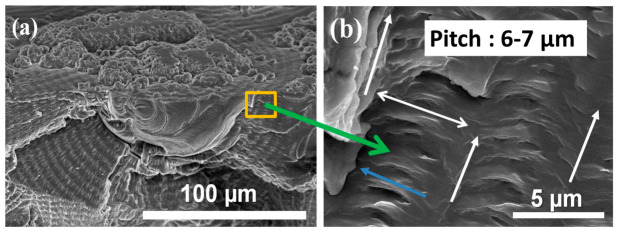
SEM micrographs revealing the lamellar assembly of ring bands adjacent to the nucleus zone in PEA spherulites. (**a**) Low-magnification view of a fractured PEA spherulite; the yellow box marks the region enlarged in (**b**). (**b**) High-magnification view of the periodic banded interior showing the corrugate-board-like lamellar assembly. The white double-headed arrow indicates the cross-bar pitch (inter-layer spacing, ~6–7 μm), corresponding to the optical inter-band spacing. White arrows indicate the radial growth direction of the lamellar layers, while the blue arrow highlights the tangential lamellar layer. The green arrow indicates the magnified region from panel (**a**).

As the fracture line of the interior may cut across various sections with random angles of the discretely layered structure, it is necessary to examine different spots for universality of the crystal assembly leading to the periodic banding patterns on top surfaces as well as in interiors. Figure 6a shows spots of the fractured interior of the same banded PEA spherulite, displaying a strut-rib (i.e., cross-hatch) grating. Figure 6b is the zoom-in SEM graph for the fractured interior (on right-hand side) of the first band neighboring the nucleus zone (the first band outside of nucleus zone), exposing the radial crystal lamellae stacks (rib-crystals). The white double-headed arrow indicates the cross-bar pitch (inter-layer spacing, ~6–7 μm), corresponding to the optical inter-band spacing. White arrows indicate the vertical growth direction of the lamellar layers, while the blue arrow highlights the horizontal lamellar layer. Again, each layer of the band is composed of the tangential and radial shells, each intersecting at approximately a 90° angle and with total layer thickness = 7 µm. Thus, the general characteristic of interior crystal assembly of the first band is proven here to be almost identical to that of the third band (counting from the rim), which proves the universality of interior crystal assembly between the inner and outer bands. On the other hand, if fracture was across different facets, the morphology along the tangential shell might differ correspondingly. The zoom-in to the fracture interior happened to locate on the inter-layer interface, thus exposing the inter-band tangential crystals (strut-lamellae) that eventually go upward to the top surface to emerge as a bulged “ridge band”.

**Figure 7:**
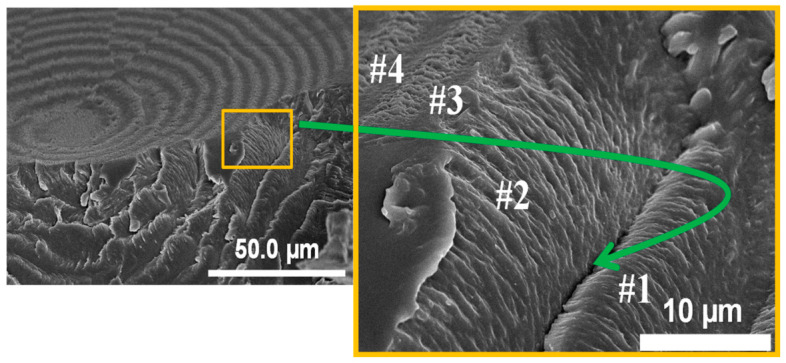
(**Left**) SEM micrograph of the fractured interior of a PEA spherulite, revealing the periodic discontinuity between successive lamellar bands. The yellow box indicates the region enlarged in the right panel. (**Right**) High-magnification SEM image of the selected region, showing the fracture surface parallel to the inter-layer interfaces (lateral view in the tangential orientation) of the corrugate-board-like lamellar architecture.

For reproducibility of results, PEA specimens were alternatively fractured for analyses on other interior locations, where the fracture traversed not along a flat plane but ladder-like descending rings in the 3D interior. Figure 7 shows SEM micrographs for fractured interior lamellae of additional PEA specimens with periodic bands crystallized at the same T_c_ = 28 °C, which reveals that the interior lamellae at various bands of increasing radius were exposed, and a ladder-like interior was the result, which again is responsible for the noted periodic banding pattern with discontinuity. Spots on our ascending ladders (#1 to #4) of successive bands are marked, where the interface between #1 and #2 layers corresponds to clean cleavage of inter-band zones upon exposure of interiors. The lateral side on #2 layer corresponds to the tangential-oriented lamellae that are spawned and branched out in each growth cycle on the interfaces where expelled amorphous impurities are concentrated. Once again, the discontinuity (marked by the green arrow) between the successive layers is obvious in the dissected interiors of the PEA-banded spherulites.

**Figure 8:**
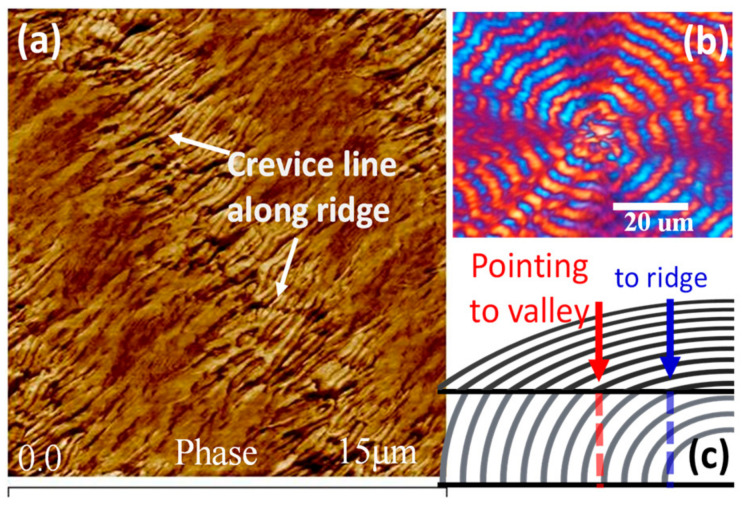
(**a**) AFM image showing apparent split boundary on top-surface banded PEA (reprinted with copyright permission) [40], (**b**) corresponding POM image, and (**c**) scheme illustrating periodic patterns corresponding to detachment in interior layers (each ~7–8 µm thick) that can be cleanly cleaved in regularly crystallized bulk PEA samples (T_c_ = 28 °C).

Top-surface-relief patterns can be better discerned using AFM for comparison. Figure 8a shows an AFM zoom-in image on the top surface of regularly crystallized PEA (at T_c_ = 28 °C) until full crystallinity. For comparison, Figure 8b shows the corresponding POM with banded rings of the PEA sample prepared using the same treatments as those for SEM/AFM characterizations; the scheme in Figure 8c illustrates the periodic patterns corresponding to detachment in interior layers (each ~7–8 µm thick), which can be cleanly cleaved in regularly crystallized bulk PEA samples (T_c_ = 28 °C). The clean cleavage on the top surface between two bands suggests discontinuity between bands in the interior bulk. The AFM zoom-in image for top-surface morphology of double ring-banded spherulite of PEA film crystallized at 28 °C (as crystallized for 30 min) reveals typical alternate ridge (higher fibrous region) vs. valley (lower flat region) bands that are easily discernible in the AFM image. However, with closer observation, one notices that there is a narrow “split line”, i.e., an interface on the middle line of the ridge that appears to be slightly zig–zag but roughly on the centerline of the ridge. This evidence likely hints that the “ridge” might be an interface or impingement of two oppositely oriented lamellae in the bulk interiors underneath the top-surface banding. The AFM result for top-surface patterns reveals the same discontinuity in inter-band interfaces as that found in the SEM results for interiors discussed earlier. Later analysis will focus on probing the origin and mechanism of this discontinuous interface between bands.

### 3.3. Correlation Between Top-Surface Banding vs. Interior-Assembled Periodicity

**Figure 9:**
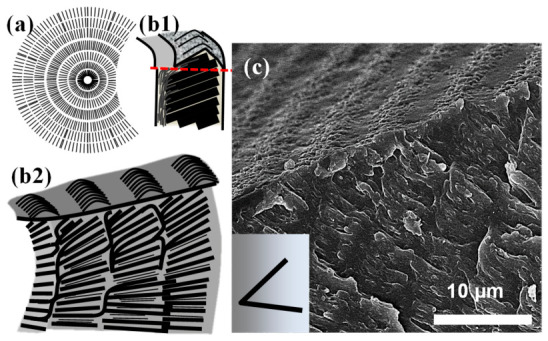
Top-surface bands on interior onion-like shells: (**a**,**b1**,**b2**) schemes for tangential and radial lamellae underneath the top-surface bands, the red dashed line denotes the interface between the top-surface lamellae and the underlying interior lamellar assembly, (**c**) SEM graph for interior lamellae assembly.

The fractured interior of ring-banded PEA spherulites, as shown in Figure 9, clearly demonstrates that the fibril tangential crystals go vertically up to the top surface, bend slightly upon reaching the top, and merge at the beginning of the ridge band. Once reaching the top surface, these fibril crystals bend and tilt toward the radial direction, and thus appear as concave-shaped fibril textures to shape the ridge band (of ca. 4–5 µm in width, decreasing with respect to distance from the nuclei core). The interior radial crystals, on the other hand, are stacked directly underneath the valley band of the top surfaces, with the valley band appearing as flat or convex-shape gaps of featureless textures (width of valley band ~1–2 µm, increasing with respect to distance from nuclei core). Thus, when a ring-banded spherulite is viewed from the top surface, the morphology may differ from that viewed from the fractured lateral sides. The top-surface-banded morphology displays an alternating concave-shape fibril-textured ridge band interspersed with featureless convex-shape valley gaps. The lateral crystal morphology of the ring-banded spherulites, as viewed from the fractured exposure, displays an alternating corrugated-board stacking. Such stacking is similar to a multi-layer shish-kebab structure, where the tangential fibril crystals act as the shish sub-structure and the radial crystals (perpendicularly grown to or from the tangential crystals) serve as the kebab sub-structure. In sum, the SEM evidence reveals that the thickness of the total shish + kebab layers is ca. 6.7–7.0 µm, which is the inter-band spacing as viewed in the POM results. Therefore, if one ignores the regions of inter-spherulite impingement upon full growth, from a low-magnification SEM graph, the dissected SEM result would have shown that the grating structure resembles a cross-cut onion with distinct shells mutually wrapped and stacked.

**Figure 10:**
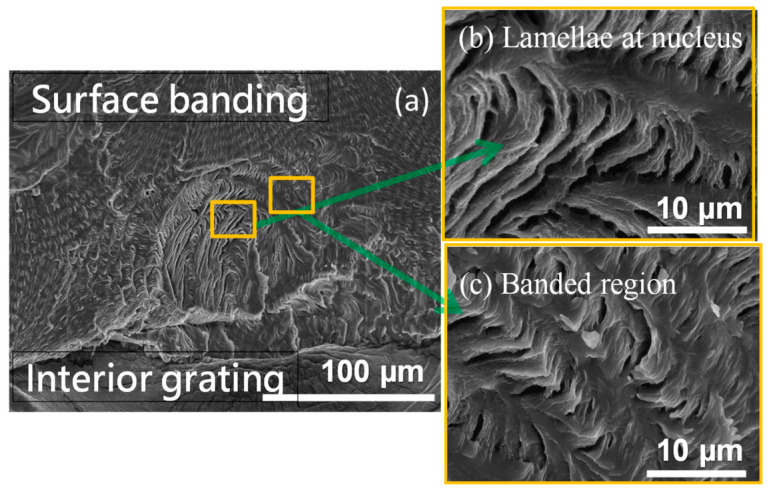
SEM micrographs of the top-surface and interior architectures of a banded PEA spherulite. (**a**) Low-magnification view revealing the top-surface banding (upper) and the fractured interior grating (lower). The yellow boxes indicate the regions enlarged in panels (**b**,**c**). The green arrows show the correspondence between the selected regions in (**a**) and their respective magnified SEM images.

Figure 10a shows a low-magnification image of the gross top surface of a banded PEA with contrasting top surface (upper left) and portions of the interior (lower right); Figure 10b displays zoom-in SEM images to the lamellae at inner cores near the nucleus; Figure 10c shows the exterior bands farther away from the nucleus but closer to the top surface, both displaying periodic grating architectures. The interior cross-hatch grating lamellae display periodic up-and-down feature just like that seen on top surface bands. The interior inter-band shell thickness (cross-bar pitch) exactly matches with the optical inter-band spacing = ca. 6–7 µm. That is, the top bands have the same inter-band spacing as those of the inter-layers in interiors.

**Figure 11:**
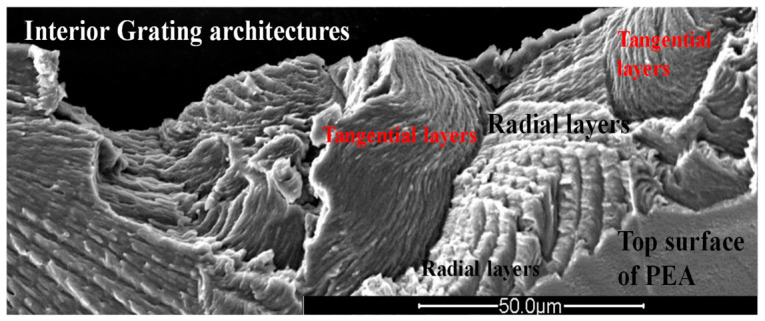
Zoom-in SEM micrograph for corrugate-board grating-structure in banded PEA spherulites (T_c_ = 28 °C), with distinct discontinuity between the tangential- and radial-oriented layered crystals (lamellar bundles), the red text highlights the tangential lamellae.

To further justify the results, dissected microscopic images in a broad range of repetitive periodicity are analyzed to reveal the distinct alternate-layered bundles of lamellae self-assembled in grating patterns similar to corrugated-boards. Figure 11 shows a full-range SEM image of fractured interiors, which further proves that as fracture/dissection was statistically across all possible faces of the bulk interior, multiple-stacked shells are visible in SEM images. The fracture happened to be across the tangential faces and radial lateral faces of the interior. This usual happenstance granted a chance to clearly view the entire discontinuous inter-band interface and periodicity along the radial direction. Apparently, the SEM microscopy characterization on the interior fracture dissection has again proven the same results as those shown and discussed in previous figures. Experimental evidence in this work is clear that distinct interfacial boundaries exist between successive layers of lamellae (which appear on top surface as concentric rings) along the radial direction. The SEM morphology data well match with the X-ray microbeam analysis as reported earlier in the literature for the alternate-grating assembly in PEA spherulites [41,42].

Universal analogy of interior cross-intersection with those in other banded polymer crystals is seen in other polymeric materials too. To advocate the observations in the banded PEA as universal features, X-ray analysis via microbeam techniques on microdomains of PEA was performed in an earlier work [42]. In dissected morphology, the same features in other polymers, probed in earlier work, are mentioned here as additional evidence. A relevant earlier study on poly(dodecamethylene terephthalate) (abbr. code name: PDoT or P12T) spherulites melt-crystallized at 96 °C reveals the same features [43,44]. P12T specimens were fractured in similar methods as that for PEA, and characterized by SEM microscopy techniques. This fact strongly contests the classical propositions that periodic bands in polymers are a consequence of continuous helical twist lamellae radiating outward from the nucleus. Universal analogies of interior cross-intersections similar with PEA can be found in other banded polymer crystals too. Thus, interior discontinuous layers in banded PEA are not just an incidental or occasional happenstance case. Discontinuity and interfacial boundaries exist not only in PEA of this study as proven above; they are also universal features, appearing in many other polymers crystallized with periodic rings.

## 4. Conclusions

Universally, interior-dissected PEA spherulites are expounded to display periodic grating assembly as viewed in POM images showing periodic bands; the interior lamellae of inner bulk actually emerge in periodic spacing from substrate to the top surface to create the ridge portion of the bands. Therefore, the angle between these protruded crystals with respect to the top planar surface is regarded as highly influencing the width and roughness of the ridges as the slant angles of tangential lamellae protrusion are related to the ways of lamellae bending on reaching the top surface. These delicately dissected morphology results are in full agreement to the POM results showing alternate optical birefringent bands and the inner cross-bar pitch perfectly matches with the optical inter-band spacing. From these analyses on interiors and top-surface patterns in the banded PEA spherulites, the discontinuous corrugate-board structure (with 7 µm layer thickness) is a better mechanism for the hierarchically structured PEA spherulites with periodic bands (typically at T_c_ = 28 °C).

Rigorous re-examination in deeper scopes for plausibility evidently confirms that it may not be essential or a prerequisite for periodically banded assemblies to be composed of continuous helical twist lamellae (i.e., 360° helical rotation like those in well-known DNA double helices). Discontinuity due to abrupt change of lamellar orientation appears to be a universal feature present in many other periodic-banded crystals. Dissected PEA morphology obviously is in excellent correlation with its optical birefringence patterns (grating layer thickness in SEM equal exactly to the inter-band spacing in POM). The general features of surface-relief patterns for banded PEA in films are matched with the interior lamellae in 3D views for 28 –crystallized PEA, illustrating the differentiation from the top-surface to interior lamellae in a typical crystallized PEA film sample. Furthermore, evidence-justified interpretations and deeper probing of mechanisms are validly demonstrated.

## Figures and Tables

**Table 1:** Sample preparations for various PEA sample film thicknesses.

Concentration	Thickness (µm)
8 wt.%, 3 drops	45–55
2 wt.%, 3 drops	20–30
8 wt.%, 1 drop	10–20
2 wt.%, 1 drop	3–10

## Data Availability

The original contributions presented in this study are included in the article. Further inquiries can be directed to the corresponding author.
